# Effects of different treatments on metabolic syndrome in patients with obstructive sleep apnea: a meta-analysis

**DOI:** 10.3389/fmed.2024.1354489

**Published:** 2024-03-07

**Authors:** Jianing Liu, Jiahuan Xu, Shibo Guan, Wei Wang

**Affiliations:** Institute of Respiratory and Critical Care Medicine, The First Hospital of China Medical University, Shenyang, Liaoning, China

**Keywords:** obstructive sleep apnea, metabolic syndrome, continuous positive airway pressure, exercise, diet, surgery, meta-analysis

## Abstract

**Background:**

Obstructive sleep apnea (OSA) and metabolic syndrome (MetS) often coexist, and the causal relationship between them is not yet clear; treatments for OSA include continuous positive airway pressure (CPAP), mandibular advancement device (MAD), surgery, and lifestyle intervention and so on. However, the effects of different treatments on metabolic syndrome in OSA patients are still under debate.

**Objectives:**

Review the effects of different treatments on metabolic syndrome in OSA patients by meta-analysis.

**Methods:**

we searched articles in PubMed, Embase, Cochrane Library, CNKI, CBM, and Wanfang data from database construction to Feb. 2024.RevMan5.4 and Stata software were used to conduct a meta-analysis of 22 articles.

**Results:**

A total of 22 articles were finally included. The results showed that CPAP treatment could reduce the prevalence of metabolic syndrome in OSA patients in randomized controlled trials (RCTs) (RR = 0.82 [95% CI, 0.75 to 0.90]; *p* < 0.01) and single-arm studies (RR = 0.73 [95% CI, 0.63 to 0.84]; *p* < 0.01). As for metabolic syndrome components, CPAP treatment reduces blood pressure, fasting glucose (FG), triglycerides (TG), and waist circumference (WC) but can’t affect high-density lipoprotein cholesterol (HDL-C) levels. Lifestyle intervention could significantly reduce the prevalence of metabolic syndrome in OSA patients (RR = 0.60 [95% CI, 0.48 to 0.74]; *p* < 0.01) and can lower blood pressure, fasting glucose, and waist circumference but can’t affect the lipid metabolism of OSA patients. Upper airway surgery can only reduce TG levels in OSA patients (MD = −0.74 [95% CI, −1.35 to −0.13]; *p* = 0.02) and does not affect other components of metabolic syndrome. There is currently no report on the impact of upper airway surgery on the prevalence of metabolic syndrome. No study has reported the effect of MAD on metabolic syndrome in OSA patients.

**Conclusion:**

We confirmed that both CPAP and lifestyle intervention can reduce the prevalence of MetS in OSA patients. CPAP treatment can lower blood pressure, fasting glucose, waist circumference, and triglyceride levels in OSA patients. Lifestyle intervention can lower blood pressure, fasting glucose, and waist circumference in OSA patients. Upper airway surgery can only reduce TG levels in OSA patients.

**Systematic review registration:**

https://www.crd.york.ac.uk/PROSPERO/, identifier CRD42022326857.

## 1 Introduction

Obstructive sleep apnea is a common disease characterized by repetitive airway collapse during sleep, which can lead to decreased blood oxygen saturation, causing chronic intermittent hypoxia (CIH) and sleep fragmentation. The main symptoms include snoring, sleep apnea, and daytime sleepiness (1). MetS is a common comorbidity of OSA; the main diagnostic criteria include abdomen obesity, hypertension, hyperglycemia, and dyslipidemia (2). A growing number of studies have shown that OSA is associated with an increased risk of MetS (3, 4). As the leading cause of metabolic disorders, CIH can promote sympathetic nerve activation, thus increasing blood pressure (5). CIH increases triglycerides and phospholipid synthesis, inhibits liver cholesterol uptake, and reduces triglycerides clearance, thus contributing to hyperlipidemia (6–8). As for glucose metabolism, CIH induces insulin resistance by causing β cell dysfunction and adipose tissue inflammation (9–11). Among adults aged 30 to 70, approximately 13% of men and 6% of women have moderate to severe OSA (12). Studies have shown that the prevalence of MetS in the Chinese population over 15 years old is 24.5% (13). It is estimated that 50–60% of obese people with MetS have OSA. Many previous studies have shown that both the prevalence of OSA in patients with MetS and the prevalence of MetS in OSA patients were at a high level (between 60 and 70%) (14–16), and both OSA and MetS were associated with an increased risk of cardiovascular disease.

The overlap in health outcomes in individuals with MetS and OSA makes it difficult to disentangle cause and effect and whether OSA treatments, such as CPAP, MAD, upper airway surgery, and weight loss, can improve these outcomes (17). As the first-line treatment for moderate and severe patients with OSA (18), CPAP treatment can effectively reduce the collapse of the upper airway, alleviate hypoxia, and improve clinical symptoms, including snoring and excessive daytime sleepiness (9). The randomized controlled trials conducted by Giampa et al. (19, 20) showed a significant decrease in the incidence of MetS in OSA patients after CPAP treatment compared to the control group. However, other studies have reached the opposite conclusion, suggesting that CPAP did not change the prevalence of MetS (21, 22). Among the various components of MetS, previous studies have only reached a consensus on reducing blood pressure after CPAP treatment (23). There were many controversies regarding its impact on other components of MetS, such as fasting glucose, blood lipids, and obesity. Upper respiratory surgery is a standard treatment for OSA. It is suitable for OSA patients who cannot tolerate CPAP or have apparent upper respiratory tract structural abnormalities. Traditional surgical methods include Uvulopalatopharyngoplasty (UPPP) and related soft tissue procedures, maxillomandibular advancement, tracheostomy (rarely used), and hypoglossal nerve stimulation. Upper airway surgery can reduce the apnea and hypopnea index of OSA patients, thereby improving their quality of life. Surgical intervention is an irreplaceable way to improve OSA for patients with severe OSA who are complicated with anatomical abnormalities (24). Surgery undoubtedly can improve the condition of OSA, but its improvement of metabolic disorders complicated with OSA was controversial in previous studies. The study of Warner et al. (25) proved that upper airway surgery can reduce blood pressure and fasting glucose levels in OSA patients but can’t affect the reduction of blood lipids. However, some studies have suggested that upper airway surgery can significantly reduce triglyceride levels in OSA patients (26), while previous studies have failed to report the impact of surgery on the prevalence of MetS. Lifestyle intervention for OSA patients includes a low-calorie diet and exercise training. Lifestyle intervention can improve the severity of OSA, improve the co-existing metabolic disorders in patients, and reduce the risk of obesity, hypertension, and diabetes (27–29). Lifestyle intervention is recommended for obese OSA patients of any severity. It can be used as a primary treatment and combined with other therapies (18). MAD applies to patients with mild and moderate OSA. Some studies have shown that applying MAD can reduce patients’ blood pressure but does not affect fasting glucose and blood lipids (30, 31). There is no study report on the effect of MAD on the prevalence of MetS in OSA patients. As for the impact of different MetS treatments on OSA patients, there is heterogeneity among the results of previous studies. In this study, we conducted a meta-analysis to investigate whether rational treatment of OSA improves the co-existing MetS in patients.

## 2 Methods

### 2.1 Search strategy

We systematically searched English articles using PubMed, Embase, and the Cochrane Library and searched the Chinese ones using CNKI, CBM, and Wanfang data. The keywords used for the search included “Metabolic Syndrome,” “obstructive sleep apnea,” “sleep-disordered breathing,” “OSA,” “OSAHS,” and corresponding Chinese words. The search time of studies was from the time of database construction to Feb.2024.2 Independent reviewers performed the study searching and screening process, and disagreements were resolved by discussion.

### 2.2 Inclusion and exclusion criteria

The inclusion criteria were: (1) published articles on OSA patients combined with MetS; (2) only adults (aged 18 years) were included; (3) CPAP, surgery, or lifestyle intervention was applied, and the duration of CPAP therapy was ≥2 weeks; and (4) Reported the number of patients with MetS or one of the following indicators before and after treatment: triglycerides, high-density lipoprotein cholesterol, blood pressure, fasting glucose, and waist circumference;

The following studies were excluded: (1) Reviews, abstracts, case reports, letters, and non-human studies. (2) Insufficient information provided.

### 2.3 Quality assessment

Two independent reviewers (J. N. Liu and J. H. Xu) evaluated the included studies using the quality assessment method, and disagreements were resolved via discussion. The Cochrane Collaboration for Systematic Reviews of Interventions was used to assess randomized controlled trials, the methodological index for non-randomized studies (MINORS) was used to evaluate non-randomized clinical trials, and the Newcastle-Ottawa Scale (NOS) was used to assess the quality of single-arm studies. Agreements between reviewers were resolved by discussion to reach a consensus.

### 2.4 Data extraction

We extracted data from 22 studies. These data were the first author, year of publication, study design, body mass index (BMI), AHI or RDI values of the participants, age, number of subjects, the duration of intervention, the number of patients with MetS, the levels of TG, HDL-C, SBP, DBP, fasting glucose and waist circumference, data from both the experimental groups and control groups in controlled trials, before and after treatment in single-arm studies. Our final analysis examined the differences in these values between them. Two independent reviewers performed this process.

### 2.5 Data synthesis

We used mean differences (MDs) and standard deviations for continuous data such as TG, HDL-C, SBP, DBP, fasting glucose, and waist circumference. RR was used to estimate the impact of different treatments on the prevalence of MetS. The associated CIs were calculated with *p*-values < 0.05, indicating significance. We also use funnel plots and Egger’s test to assess publication bias. Heterogeneity was assessed using the Cochrane Q and chi-square tests with I-squared index tests. I-square >0% with *p*-values < 0.05 was considered high heterogeneity, using the random-effects model; otherwise, the fixed-effects model was used. Cochrane Collaboration’s RevMan 5.4 and Stata software were used to analyze all data.

## 3 Results

**FIGURE 1:**
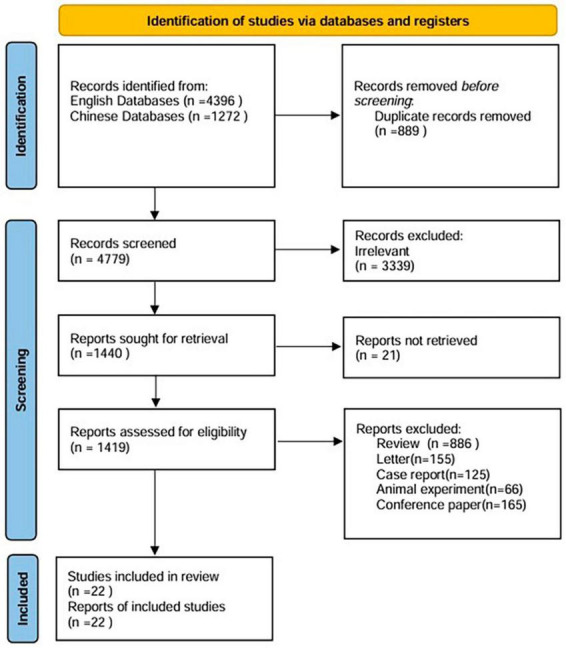
Flow diagram of the study selection process.

A total of 5,668 studies were identified from different sources. Finally, data were taken from 22 articles, including 9 Chinese and 13 English articles. Figure 1 shows the process of the literature search. The characteristics of the studies are summarized in Table 1. According to the funnel plots and Egger’s test outcome, the included studies had no significant publication bias.

**TABLE 1:** Characteristics of the enrolled studies in the meta-analysis.

References	Study design	Intervention	Population	AHI
Coughlin et al. (21)	RCT	CPAP	34	39.7 ± 13.8 (RDI)
Giampa et al. (20)	RCT	CPAP	100	58 ± 29
Sun et al. (19)	RCT	CPAP	128	CPAP: 48.9 ± 19.6 control: 46.8 ± 17.3
Hoyos (48)	RCT	CPAP	65	39.9 ± 17.7
Salord et al. (22)	RCT	CPAP	80	CPAP: 68.3 (43–88) control: 52.6 (37–78)
Xiao et al. (32)	RCT	CPAP	158	CPAP: 14 ± 12 control: 15 ± 10
Mota (49)	Single-arm study	CPAP	47	46.9+33.6
Oktay (50)	Single-arm study	CPAP	30	54.8 ± 22.9
Li et al. (6)	Single-arm study	CPAP	52	37.67 ± 17.95
Mineiro (51)	Single-arm study	CPAP	34	35.2 ± 18.8
Dorkova (52)	Single-arm study	CPAP	32	64.0 ± 20.8
Oyama (53)	Single-arm study	CPAP	32	56.2 ± 21.6
Xia (54)	Single-arm study	CPAP	112	–
Guo (55)	Single-arm study	CPAP	40	–
Desplan (56)	RCT	Exercise	20	Experimental 40.6 ± 19.4 control 39.8 ± 19.2
Cristiane (57)	Clinical trials	Low-calorie diet+exercise	24	Experimental 36 ± 5.1 control 36 ± 10.1
Edgar (58)	Clinical trials	Low-calorie diet+exercise	23	Experimental 31 ± 5 control 37 ± 7
Georgoulis et al. (27)	RCT	Low-calorie diet+exercise	180	MDG: 62 (37, 81) MLG: 60 (21,89) control: 52 (30, 84)
Peng (59)	Single-arm study	Surgery	45	55.1 ± 17.8
Hua (60)	Single-arm study	Surgery	23	51.9 ± 15.8
Zhu (61)	Single-arm study	Surgery	35	41.1 ± 6.2
Chen et al. (5)	Single-arm study	Surgery	26	37.65 ± 15.59
**References**	**BMI**	**Age**	**Duration of trials**	**Outcome**
Coughlin et al. (21)	36.1 ± 7.6	49 ± 8.3	1.5 months,3.9 ± 0.74 h/night	SBP, DBP, HDL-C, TG, FG, Mets
Giampa et al. (20)	33 ± 4	48 ± 9	6 months,5.5 ± 1.5 h/night	SBP, DBP, HDL-C, TG, FG, WC, Mets
Sun et al. (19)	CPAP 33.8 ± 4.7 control 31.8 ± 5.2	44 ± 9	5 months	SBP, DBP, HDL-C, TG, FG, WC, Mets
Hoyos (48)	31.3 ± 5.2	49 ± 12	3 months, CPAP 3.6 h/night,control 2.8 h/night	SBP, DBP, FG, WC, Mets
Salord et al. (22)	CPAP 45.7 ± 5 control 49.3 ± 6.6	CPAP 48.5 ± 8.6 control 44.6 ± 9.4	3 months,5.4 ± 1.6 h/night	SBP, DBP, HDL-C, TG, FG, WC, Mets
Xiao et al. (32)	CPAP 27.2 ± 2.19 control 27.1 ± 2.3	–	1 month	SBP, DBP, HDL-C, TG, FG
Mota (49)	33.4 +8.4	55.9 (10.7)	6 months	SBP, DBP, HDL-C, TG, FG, WC, Mets
Oktay (50)	32.9 ± 4	50.5 ± 7.7	12 months, >6 h/night	SBP, DBP, HDL-C, TG, FG, WC, Mets
Li et al. (6)	29.56 ± 2.36	–	3 months	SBP, DBP, HDL-C, TG, FG, WC, Mets
Mineiro (51)	31.2 ± 4.1	55.2 ± 7.9	4 months, ł 4 h/night	SBP, DBP, HDL-C, TG, FG, Mets
Dorkova (52)	35.1 ± 6.1	53.7 ± 9.6	2 months	SBP, DBP, HDL-C, TG, FG
Oyama (53)	26.7 ± 3.6	54 ± 9	3 months, ł 4 h/night	SBP, DBP, HDL-C, TG, FG, WC
Xia (54)	32.41 ± 4.53	–	2 months	SBP, DBP, HDL-C, TG, FG, WC
Guo (55)	–	–	3 months, 4-6 h/night	SBP, DBP, TG, FG, WC
Desplan (56)	Experimental 29.9 ± 3.4 control 31.3 ± 2.5	35–70	1 month	SBP, DBP, HDL-C, TG, FG, WC, Mets
Cristiane (57)	Experimental 32 ± 0.7 control 32 ± 1.3	Experimental 53 ± 1.7 control 42 ± 2.6	4 months	SBP, DBP, HDL-C, TG, FG, WC, Mets
Edgar (58)	32 ± 1	Experimental 52 ± 2 control 48 ± 3	4 months	SBP, DBP, HDL-C, TG, FG, WC, Mets
Georgoulis et al. (27)	MDG 34.8 ± 5.9 MLG 35.5 ± 5.6 control 35.8 ± 6.3	MDG 51 ± 9 MLG 47 ± 10 control 47 ± 11	12 months	SBP, DBP, HDL-C, TG, FG, WC, Mets
Peng (59)	28.1 ± 3.2	41.5 ± 8.7	12 months	HDL-C, TG, FG
Hua (60)	30.7 ± 3.2	40.3 ± 9.8	6 months	SBP, DBP, HDL-C, TG, FG, WC
Zhu (61)	31.1 ± 2.3	51.4 ± 7.3	6 months	SBP, DBP, HDL-C, TG, FG, WC
Chen et al. (5)	33.73 ± 4.28	–	6 months	SBP, DBP, HDL-C, TG, FG, WC

### 3.1 Qualitative analysis

All included articles were published from 2007 to 2023. Of the 22 articles, 8 were RCTs, 2 were non-randomized controlled clinical trials, and the other 12 were single-arm studies. The mean age ranged between 35 and 70. The average Body Mass Index (BMI) ranged between 26.7 and 49.3 kg/m^2^. The mean AHI ranged between 14 and 68.3/h. The duration of CPAP treatment and lifestyle intervention was 4 weeks to 12 months. The evaluation of surgical effectiveness is 6 or 12 months later.

### 3.2 Quality assessment

**FIGURE 2:**
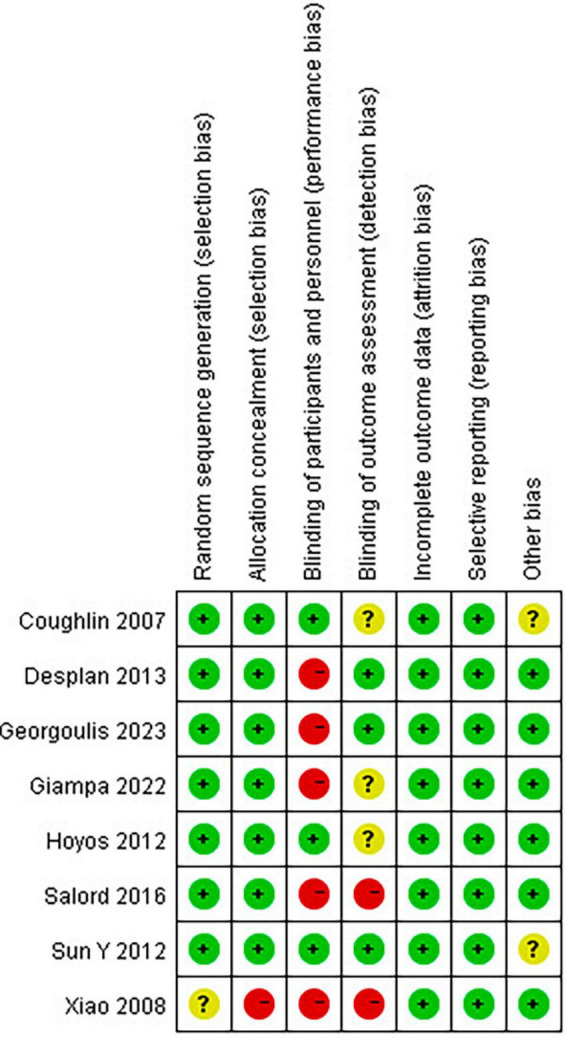
Risk of bias summary: review authors’ judgments about each risk of bias item for each included study.

Newcastle-Ottawa Scale was used to evaluate the quality of single-arm studies. The eight single-arm studies’ scores were six points or above, indicating high research quality. The MINORS scale was used to evaluate the study quality of non-randomized clinical trials. The scores of the two non-randomized clinical trials included were 21 points, indicating high study quality. The results of the Cochrane Bias risk assessment of RCTs are shown in Figure 2.

### 3.3 Pooled analysis on MetS prevalence

#### 3.3.1 Pooled analysis of the impact of CPAP on MetS prevalence

**FIGURE 3:**
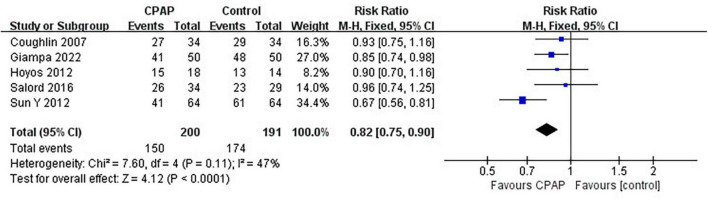
Impact of CPAP on Mets prevalence (RCTs).

**FIGURE 4:**
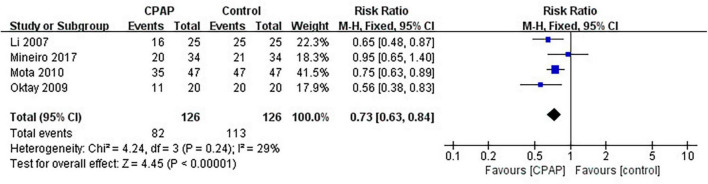
Impact of CPAP on Mets prevalence (single-arm studies).

Nine studies have reported the number of patients diagnosed with MetS before and after CPAP treatment, five of which were randomized controlled trials. Pooled meta-analysis showed that compared with untreated patients, CPAP treatment could reduce the prevalence of MetS in OSA patients in both RCTs (RR = 0.82 [95% CI, 0.75 to 0.90]; *p* < 0.01) (Figure 3) and single-arm studies (RR = 0.73 [95% CI, 0.63 to 0.84]; *p* < 0.01) (Figure 4).

#### 3.3.2 Pooled analysis of the impact of lifestyle intervention on MetS prevalence

**FIGURE 5:**
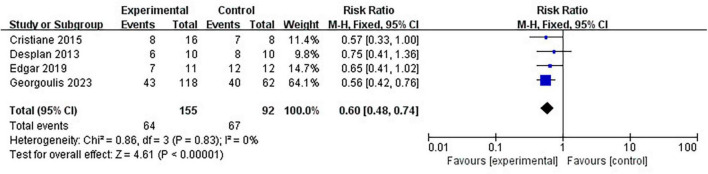
Impact of lifestyle intervention on Mets prevalence.

Four clinical trials have reported the number of patients diagnosed with MetS before and after lifestyle intervention. Pooled meta-analysis showed that lifestyle intervention could significantly reduce the prevalence of MetS in OSA patients (RR = 0.60 [95% CI, 0.48 to 0.74]; *p* < 0.01) (Figure 5).

#### 3.3.3 Pooled analysis of the impact of surgery and MAD on MetS prevalence

No study has reported the effect of surgery or MAD on MetS prevalence in OSA patients.

### 3.4 Pooled analysis of the changes in MetS components

#### 3.4.1 Pooled analysis of the impact of CPAP on SBP

**FIGURE 6:**
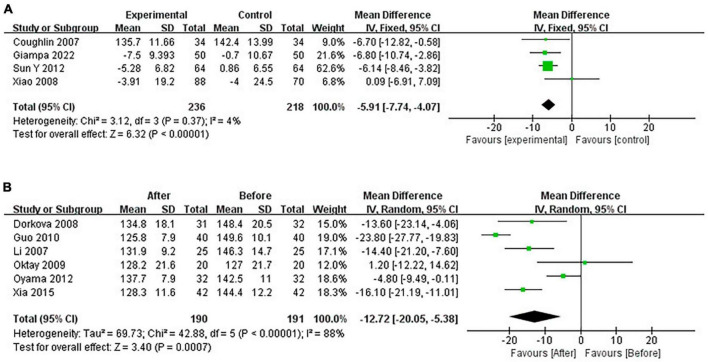
Change in SBP [**(A)**: RCTs; **(B)**: single-arm studies].

Continuous positive airway pressure reduced SBP in OSA patients. A total of 4 RCTs (MD = −5.91 [95% CI, −7.74 to −4.07]; *p* < 0.01) and 6 single-arm studies (MD = −12.72 [95% CI, −20.05 to −5.38]; *p* < 0.01) were included. The difference was statistically significant (Figure 6).

#### 3.4.2 Pooled analysis of the impact of CPAP on DBP

**FIGURE 7:**
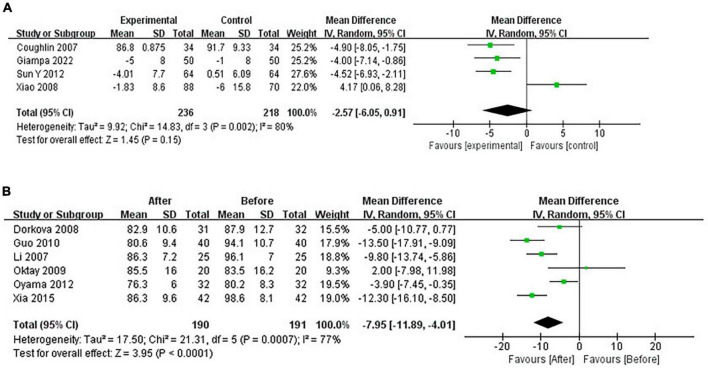
Change in DBP [**(A)**: RCTs; **(B)**: single-arm studies].

Continuous positive airway pressure reduced DBP in OSA patients. A total of 4 RCTs (MD = −2.57 [95% CI, −6.05 to 0.91]; *p* = 0.15) and 6 single-arm studies (MD = −7.95 [95% CI, −11.89 to −4.01]; *p* < 0.01) were included. The difference in single-arm studies was statistically significant (Figure 7).

#### 3.4.3 Pooled analysis of the impact of CPAP on FG

**FIGURE 8:**
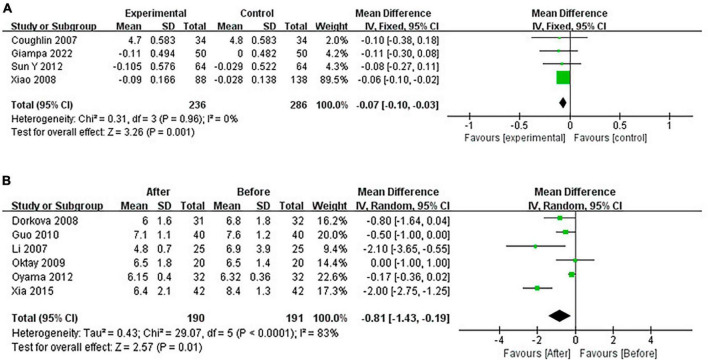
Change in FG [**(A)**: RCTs; **(B)**: single-arm studies].

Continuous positive airway pressure reduced FG in OSA patients. A total of 4 RCTs (MD = −0.07 [95% CI, −0.10 to −0.03]; *p* = 0.001) and 6 single-arm studies (MD = −0.81 [95% CI, −1.43 to −0.19]; *p* = 0.01) were included. The difference was statistically significant (Figure 8).

#### 3.4.4 Pooled analysis of the impact of CPAP on WC

**FIGURE 9:**
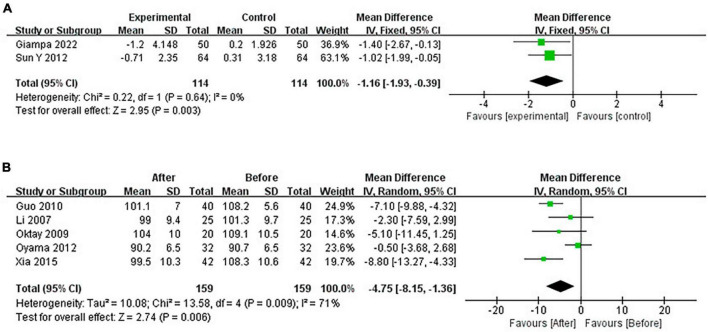
Change in WC [**(A)**: RCTs; **(B)**: single-arm studies].

Continuous positive airway pressure reduced WC in OSA patients. A total of 2 RCTs (MD = −1.16 [95% CI, −1.93 to −0.39], *p* = 0.003; *p* = 0.001) and 5 single-arm studies (MD = −4.75 [95% CI, −8.15 to −1.36], *p* = 0.006) were included. The difference was statistically significant (Figure 9).

#### 3.4.5 Pooled analysis of the impact of CPAP on TG

**FIGURE 10:**
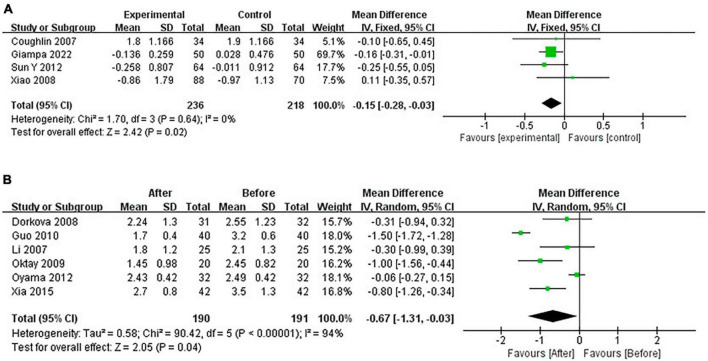
Change in TG [**(A)**: RCTs; **(B)**: single-arm studies].

Continuous positive airway pressure reduced TG in OSA patients. A total of 4 RCTs (MD = −0.15 [95% CI, −0.28 to −0.03], *p* = 0.02) and 6 single-arm studies (MD = −0.67 [95% CI, −1.31 to −0.03], *p* = 0.04) were included. The difference was statistically significant (Figure 10).

#### 3.4.6 Pooled analysis of the impact of CPAP on HDL-C

**FIGURE 11:**
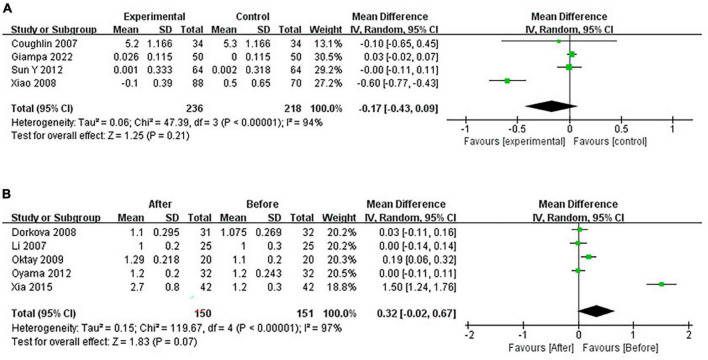
Change in HDL-C [**(A)**: RCTs; **(B)**: single-arm studies].

Continuous positive airway pressure can’t affect HDL-C levels in OSA patients. A total of 4 RCTs (MD = −0.17 [95% CI, −0.43 to 0.09], *p* = 0.21) and 5 single-arm studies (MD = 0.32 [95% CI, −0.02 to 0.67], *p* = 0.07) were included. The difference was not statistically significant (Figure 11).

#### 3.4.7 Pooled analysis of the impact of lifestyle intervention on MetS components

Lifestyle intervention could lower blood pressure, fasting glucose, and waist circumference but can’t affect the lipid metabolism of OSA patients (Table 2).

**TABLE 2:** Impact of lifestyle intervention on MetS components.

	MD [95% CI]	*P*-value	*I*^2^; *P*-value	Studies
SBP	−13.92 [−19.80, −8.04]	<0.00001	74%; 0.05	2
DBP	−4.00 [−6.19, −1.81]	0.0003	0%; 1.00	2
FG	−0.63 [−0.75, −0.50]	<0.00001	0%; 0.37	2
TG	−0.26 [−0.56, 0.04]	0.08	69%; 0.07	2
HDL-C	0.01 [−0.07, 0.08]	0.83	57%; 0.13	2
WC	−6.65 [−7.99, −5.30]	<0.00001	46%; 0.17	2

#### 3.4.8 Pooled analysis of the impact of surgery on MetS components

Upper airway surgery can only reduce TG levels in OSA patients and does not affect other components of MetS (Table 3).

**TABLE 3:** Impact of surgery on MetS components.

	MD [95% CI]	*P*-value	*I*^2^; *P*-value	Studies
SBP	−4.86 [−11.32, 1.60]	0.14	82%; 0.004	3
DBP	−4.16 [−10.82, 2.49]	0.22	89%; 0.0002	3
FG	−2.18 [−4.60, 0.23]	0.08	97%; <0.00001	4
TG	−0.74 [−1.35, −0.13]	0.02	86%; <0.0001	4
HDL-C	−0.15 [−0.31, 0.00]	0.05	80%; 0.002	4
WC	−1.03 [−4.01, 1.94]	0.50	0%; 0.95	3

#### 3.4.9 Heterogeneity and publication bias

**FIGURE 12:**
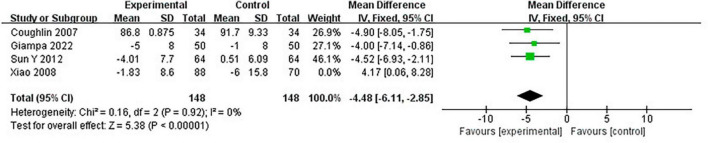
The outcome of sensitivity analysis.

Due to the heterogeneity of some included studies, this study conducted sensitivity analysis by successively excluding some studies. It was found that in the study on the effect of CPAP on DBP in OSA patients, after excluding the study of Xiao et al. (32), there is a significant change in the results (MD = −4.48 [95% CI, −6.11 to −2.85]; *p* < 0.00001) (Figure 12).

**FIGURE 13:**
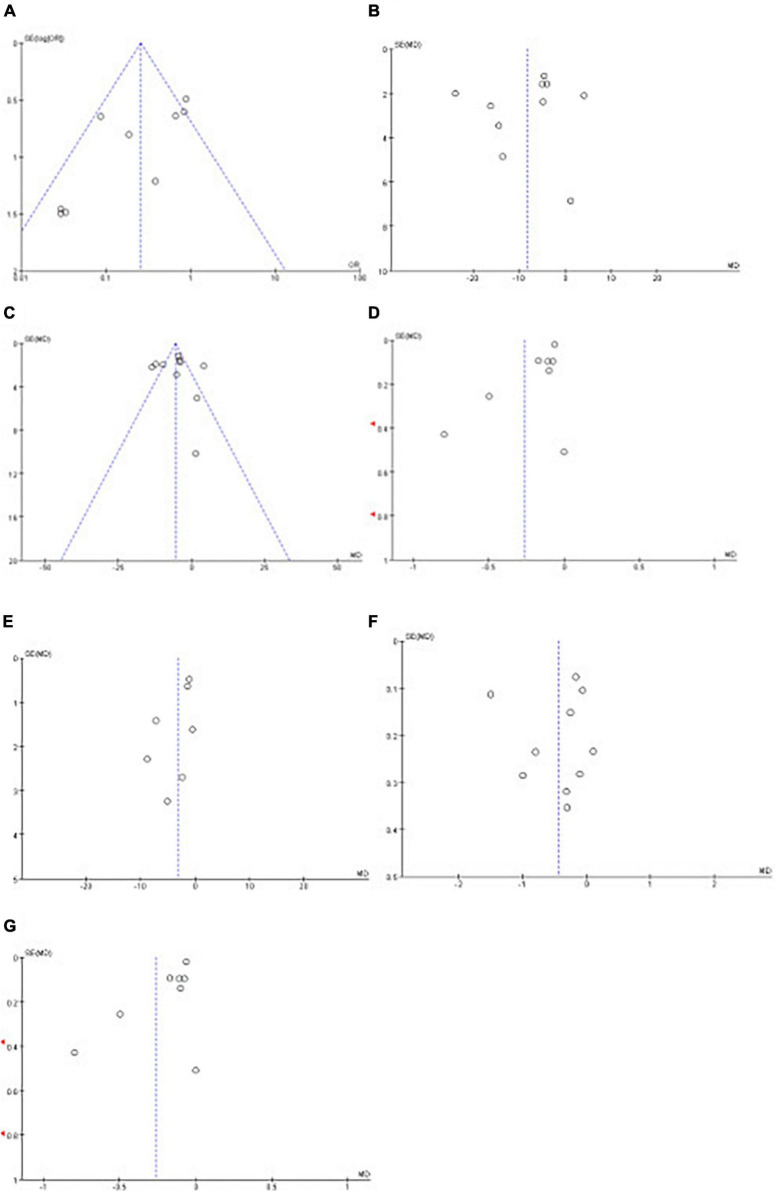
Funnel plots of the impact of CPAP. Funnel plots of the impact CPAP [**(A)**: MetS, **(B)**: SBP, **(C)**: DBP, **(D)**: FG, **(E)**: WC, **(F)**: TG, **(G)**: HDL-C].

Funnel plots were used to evaluate publication bias; if the funnel plots showed apparent asymmetry, we used Egger’s test for the quantitative evaluation. The results of Egger’s test showed that the *P*-value of the test result of CPAP’s effect on fasting blood glucose and triglyceride was <0.05, indicating the existence of publication bias. However, after trim and filling analysis, it was found that the publication bias had little impact on the study results. Publication bias was not present in the remaining outcomes. The funnel plots and Egger’s test outcome are shown in Figure 13 and Table 4.

**TABLE 4:** Egger’s test of publication bias.

Outcome	*P*-value
SBP	0.865
DBP	0.509
FG	0.021
WC	0.12
TG	0.018
HDL-C	0.645

## 4 Discussion

Previous studies have shown that OSA can promote the occurrence of MetS (3, 4, 33). CIH and sleep fragmentation mainly characterize OSA. CIH promotes the activation of the sympathetic nerves, thereby increasing blood pressure; the increased level of circulating inflammatory products and reactive oxygen results in hyperglycemia and dyslipidemia (7, 9). OSA is more common in obese patients (34). Patients with OSA are 6–9 times more likely to have MetS than the general population (35–37). The existing research on the impact of different treatments for OSA on coexisting MetS in patients has inconsistent conclusions. This study found through meta-analysis that CPAP and lifestyle intervention could reduce blood pressure, fasting glucose, and waist circumference in OSA patients, thereby reducing the prevalence of co-existing MetS. However, upper airway surgery can only reduce the triglycerides level of patients.

This meta-analysis confirmed that CPAP can reduce the prevalence of MetS, but the improvement of MetS with CPAP alone is limited (RCT: RR = 0.82; Single-arm studies: RR = 0.73). The formation of MetS is related to many factors, such as genetics, diet habits, exercise, and co-existing diseases, and OSA is only one of the many risk factors (38). In contrast, lifestyle interventions had a more dominant effect on MetS in OSA patients (RR = 0.60). Lifestyle intervention is an essential treatment for MetS (39), and the Mediterranean diet has been shown to reduce the risk of MetS and improve every component of it (40, 41). In various studies exploring lifestyle intervention, the prevalence of MetS was notably reduced in subjects receiving CPAP therapy simultaneously, suggesting that CPAP combined lifestyle intervention is essential in improving disease prognosis in OSA patients with MetS. There is an interaction between components of MetS, and weight loss can lead to changes in other components, such as blood lipid and fasting glucose (2). Therefore, lifestyle intervention is considered an essential and effective treatment for OSA (18). Our study showed that lifestyle intervention, including low-calorie diet and exercise, could reduce patients’ blood pressure, fasting glucose, and waist circumference, and the impact on waist circumference was particularly significant. Lifestyle intervention can’t affect lipid metabolism, which differs from previous studies (41, 42). This may be related to the short intervention time. The intervention time of the studies included in our report was only 4 months. Reducing blood lipid levels is a long-term process, so a short intervention may not seem to have a significant outcome. So far, no study has reported whether MAD and surgery can impact the prevalence of MetS in OSA patients.

Continuous positive airway pressure is the first-line treatment for patients with moderate to severe OSA. CPAP can effectively reduce CIH caused by airway collapse (37), but the therapeutic effect of CPAP depends on compliance. CPAP can reduce sympathetic nerve activation and vascular damage caused by intermittent hypoxia, thereby reducing blood pressure (42). Studies have proved that CPAP can significantly improve the condition of patients with refractory hypertension and can play an essential clinical role in the management of hypertension in these patients (23). Similarly, our study confirmed that CPAP can reduce blood pressure in OSA patients with MetS. There is much debate about the effects of CPAP on blood lipids in patients. Our study demonstrated a decrease in triglycerides after CPAP treatment. The formation of dyslipidemia in OSA patients with MetS is a long-term process influenced by many factors, of which OSA is only one. Triglycerides are more sensitive to reducing oxidative stress caused by OSA treatment (43), while HDL-C is mainly influenced by genetic factors and not easily changed by various intervention methods (44). When it comes to fasting glucose, we agree with previous studies. CPAP can reduce fasting glucose in OSA patients, regardless of whether they have MetS (45).

Surgery is the preferred treatment for patients who are intolerant to CPAP or have anatomic abnormalities in the upper airway. Intrapharyngeal surgery (soft tissue surgery) is widely used in treating OSA. Uvulopalatopharyngoplasty is the most effective method to solve oropharyngeal obstruction in OSA patients (18). Previous studies have confirmed the effectiveness of surgery in OSA patients (46). However, there is controversy in previous studies regarding the impact of surgery on MetS in OSA patients, and no RCTs have been published in relevant fields in the past. The studies included are all single-arm clinical trials. The meta-analysis showed that only TG was reduced after upper airway surgery. A liquid diet for some time after surgery can also reduce energy intake to some extent, thus lowering TG levels. Most of the included subjects were obese patients; after the surgery, the BMI of the patients all decreased to varying degrees, while TG levels were closely related to BMI decrease (47). All the above reasons could cause TG changes.

Our study had some limitations. First, only a few RCTs could be included, and the total number of samples was relatively small. Therefore, there were single-arm studies in our included studies. However, even so, existing studies have sufficiently confirmed the role of different treatments in reducing the incidence of MetS in OSA patients. This study analyzed RCTs and single-arm studies, respectively, and the conclusions were consistent. Secondly, in some of the studies, the duration of treatment was relatively short, which may not be sufficient to impact MetS, leading to significantly increased heterogeneity between studies. Therefore, future research requires RCTs with larger patient samples and longer follow-up times. Finally, although some studies have reported the effects of MAD on blood pressure, blood glucose, and blood lipids in OSA patients (30), no study was conducted among patients with both OSA and MetS. Therefore, this study failed to conduct a comprehensive discussion on this aspect.

In conclusion, we confirmed that both CPAP treatment and lifestyle intervention could reduce the prevalence of MetS in OSA patients. CPAP can lower blood pressure, fasting glucose, waist circumference, and triglyceride levels. Lifestyle intervention can lower blood pressure, fasting glucose, and waist circumference in OSA patients. Upper airway surgery can only reduce TG levels in OSA patients. Research suggests that for patients with OSA combined with MetS, appropriate treatment should be selected promptly based on the condition to improve the patient’s prognosis.

## Data availability statement

The original contributions presented in the study are included in the article/Supplementary material, further inquiries can be directed to the corresponding author.

## Author contributions

JL: Data curation, Formal Analysis, Methodology, Resources, Software, Writing – original draft. JX: Investigation, Resources, Supervision, Validation, Writing – original draft. SG: Data curation, Investigation, Methodology, Visualization, Writing – original draft. WW: Conceptualization, Resources, Supervision, Writing – review and editing.
